# Microporous Polylactic Acid Scaffolds Enable Fluorescence-Based Perfusion Imaging of Intrinsic In Vivo Vascularization

**DOI:** 10.3390/ijms241914813

**Published:** 2023-10-01

**Authors:** Christoph Koepple, Lukas Pollmann, Nicola Sariye Pollmann, Matthias Schulte, Ulrich Kneser, Norbert Gretz, Volker J. Schmidt

**Affiliations:** 1 Department for Hand-, Plastic- and Reconstructive Surgery, BG Trauma Center Ludwigshafen, Heidelberg University, 67071 Ludwigshafen, Germany; pollmann.lukas@yahoo.de (L.P.); nicola.roushansarai@yahoo.com (N.S.P.); matthias.schulte@bgu-ludwigshafen.de (M.S.); ulrich.kneser@bgu-ludwigshafen.de (U.K.); 2 Medical Research Center, Medical Faculty Mannheim, University of Heidelberg, 68167 Mannheim, Germany; norbert.gretz@medma.uni-heidelberg.de; 3 Department of Plastic Surgery and Hand Surgery, Kantonsspital St. Gallen, 9007 St. Gallen, Switzerland; volker.schmidt@kssg.ch

**Keywords:** fluorescence-based imaging, fluorescence microscopy, angiogenesis, tissue engineering, AV loop model

## Abstract

In vivo tissue engineering (TE) techniques like the AV loop model provide an isolated and well-defined microenvironment to study angiogenesis-related cell interactions. Functional visualization of the microvascular network within these artificial tissue constructs is crucial for the fundamental understanding of vessel network formation and to identify the underlying key regulatory mechanisms. To facilitate microvascular tracking advanced fluorescence imaging techniques are required. We studied the suitability of microporous polylactic acid (PLA) scaffolds with known low autofluorescence to form axial vascularized tissue constructs in the AV loop model and to validate these scaffolds for fluorescence-based perfusion imaging. Compared to commonly used collagen elastin (CE) scaffolds, the total number of vessels and cells in PLA scaffolds was lower. In detail, CE-based constructs exhibited significantly higher vessel numbers on day 14 and 28 (d14: 316 ± 53; d28: 610 ± 74) compared to the respective time points in PLA-based constructs (d14: 144 ± 18; d28: 327 ± 34; each *p* < 0.05). Analogously, cell counts in CE scaffolds were higher compared to corresponding PLA constructs (d14: 7661.25 ± 505.93 and 5804.04 ± 716.59; d28: 11211.75 + 1278.97 and 6045.71 ± 572.72, *p* < 0.05). CE scaffolds showed significantly higher vessel densities in proximity to the main vessel axis compared to PLA scaffolds (200–400 µm and 600–800 µm on day 14; 400–1000 µm and 1400–1600 µm on day 28). CE scaffolds had significantly higher cell counts on day 14 at distances from 800 to 2000 µm and at distances from 400 to 1600 µm on day 28. While the total number of vessels and cells in PLA scaffolds were lower, both scaffold types were ideally suited for axial vascularization techniques. The intravascular perfusion of PLA-based constructs with fluorescence dye MHI148-PEI demonstrated dye specificity against vascular walls of low- and high-order branches as well as capillaries and facilitated the fluorescence-based visualization of microcirculatory networks. Fluorophore tracking may contribute to the development of automated quantification methods after 3D reconstruction and image segmentation. These technologies may facilitate the characterization of key regulators within specific subdomains and add to the current understanding of vessel formation in axially vascularized tissue constructs.

## 1. Introduction

Within the field of regenerative medicine and tissue engineering (TE) various biologically derived acellular dermal substitutes are currently used as scaffolds for artificial soft-tissue constructs [1]. These scaffolds are designed to provide distinct characteristics for regulating microenvironments and to facilitate tissue growth and functional differentiation of cells. They orchestrate and support a multitude of biological processes involved in early tissue formation, like cell migration, attachment, and proliferation, and thereby promote tissue regeneration [2]. Serving as a substrate for cell adherence, these scaffolds are also crucial for local nutrient supply and vascularization, as well as tissue and cell growth [3]. These complex processes can be regulated and maintained by extrinsic modulation and vascularization strategies. The gradual vascularization of in vivo transplanted synthetic scaffolds from peripheral vessel sprouts is, however, only viable for smaller constructs. Further modulation either by adding bioactive stimulators, like vascular endothelial growth factor (VEGF) and basic fibroblast growth factor (bFGF), or by modifying scaffold porosity might push these limits [4,5]. However, with regard to a potential clinical application, larger constructs cannot be sufficiently vascularized by extrinsic strategies as the innermost parts of these constructs are not accessible for vascularization in a proper timeframe that simultaneously prevents the degradation and necrosis of these areas [6,7].

Functional microvascular networks within artificial tissue constructs are either predesigned or originate from an intrinsic angioinductive vascular carrier within the construct. These constructs feature a fully functional microcirculatory system, originating from an axial blood supply and can directly be transferred to a site of demand once connected to a recipient’s vessels by means of standard microsurgical techniques [8]. Complementary effects of tissue hypoxia and hemodynamic forces within a biological reactor are responsible for axial vascularization approaches [9]. This form of de novo angiogenesis is unique and enables novel therapeutic opportunities not only for TE approaches, but also for peripheral vascular diseases. Usually, a venous graft is inserted between an arteriovenous shunt (AV loop model) and embedded in an isolated subcutaneous chamber filled with an acellular dermal substitute. Vascular shear stress within the AV loop is responsible for the endothelial transactivation of Connexin43 [10], which likely triggers vessel sprouting independently of gap junction communication and independently of extrinsic bioactive factors [11].

As we begin to understand the underlying molecular pathways of this promising form of in vivo tissue and vessel formation, advanced and high-resolution optical 3D visualization techniques are required to adequately analyze the spatial and temporal coordination of key regulators. Microcirculatory networks and vascular endothelium of low or high order branches as well as capillaries have not been adequately visualized by fluorescence-based perfusion imaging within artificially engineered tissue constructs prior to this study. Histomorphometrical vessel quantification in artificial tissue constructs is usually performed after the India ink-perfusion of specimens and based on manual or automated 2D assessments [12]. Serial histological sections and scans followed by a 3D reconstruction are limited with regard to advanced vessel analysis as artefacts, and the loss of anatomical information hampers vessel-tracking [13]. Recent investigations in scaffold designs have come a long way, and increased the capacity to form functional organoid-like constructs and to mimic complex tissue types [14]. Although these developments are generally beneficial in a translational manner, they are often disadvantageous for high-resolution and functional fluorescence imaging techniques. High amounts of background signals and auto-fluorescence appears often due to the used heterogeneous and dense extracellular compositions [15,16], and high amounts of light absorption interferes with fluorescence imaging and the 3D analysis of specific subdomains within the construct. Therefore, the present study compared a homogenous microporous polylactic acid matrix (PLA) matrix with low autofluorescence [17,18] for shear stress-induced axial vascularization against a proven collagen–elastin (CE) matrix. The validation of neovessel formation, cell infiltration and matrix maturation was performed after the India ink-perfusion of the animals. We then used MHI148-PEI for the fluorescence-based intravascular perfusion of PLA-based constructs and studied its suitability for the fluorescence-based high-contrast imaging of vascular endothelium to set the stage for further 3D fluorescence imaging of microcirculatory networks within intrinsic vascularized tissue in the future. 

## 2. Results

### 2.1. Experimental Setup, Basic Data and Patency Rates upon Explantation among the Studied Groups

**Figure 1:**
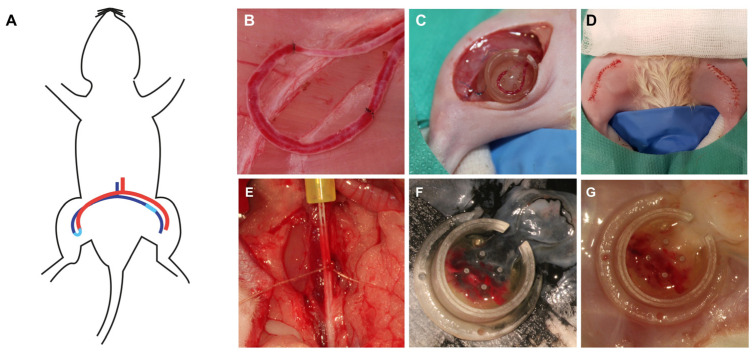
Experimental setup and AV loop procedure. Schematic representation of the AV loop operation where the femoral vein harvested from the left leg is interpositioned as a vessel graft between the contralateral vein and artery, resulting in an arteriovenous shunt (**A**). Picture taken directly after opening the blood flow of the AV loop. Microsurgical knots bordering the implanted venous graft on both sides (**B**). The chamber was sutured onto the adductor fascia, and the AV loop was embedded in two layers of CE or PLA matrix (**C**). After placement of the chamber lid, bilateral wound closure was performed (**D**). After a given timeframe the animals were reperfused with India ink (**F**) or MHI148-PEI (**E**,**G**) after cannulization of the aorta and a washing step with a diluted heparin solution.

**Figure 2:**
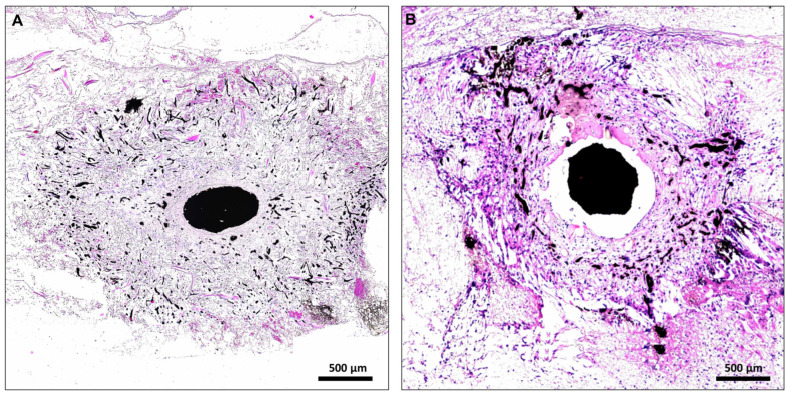
Exemplary HE-stained histological cross sections of PLA- (**A**) and CE-based (**B**) tissue constructs on day 14. CE-based constructs (**B**) were larger in size with denser soft-tissue formations apparent. Conversely, PLA-based constructs appeared more homogenous (**A**) with a looser appearance of the fibrous tissue. In both scaffolds, staining of the main vessel axis, branching vessels and peripheral capillaries were detected, revealing a functional 3-dimensional vascular network formation.

All rats tolerated the anesthesia and surgical procedures well (*n* = 35). There were no surgical site infections, hematomas or wound dehiscences. 33 animals were perfused with India ink for a histomorphometrical and histological comparison of lactocapromer–terpolymer (*n* = 8 for day 14 and 28, respectively) and collagen–elastin-based constructs (*n* = 8 and *n* = 9 for day 14 and 28, respectively). We observed a total number of 13 AV loop thromboses, which were excluded from further analysis (3 loop thromboses on day 14 for both scaffold types, respectively; 3 on day 28 of the lactocapromer–terpolymer scaffold and 4 on day 28 on collagen–elastin-based constructs). After India ink reperfusion, the isolation chamber was explanted, and the artificial construct was assessed macroscopically. Tissue formed in the isolation chamber and around the AV loop had a general milky appearance (Figure 1). 2 animals (7 and 28 days after AV loop creation) were perfused with MHI148-PEI to validate the suitability of the PLA-based scaffolds for fluorescence imaging techniques. A homogenous appearance was more pronounced in constructs loaded with PLA-based scaffolds. Macroscopically and microscopically, tissue and cell density were higher in constructs loaded with collagen–elastin-based scaffolds (compare Figure 2).

### 2.2. Comparative Histomorphometrical Studies of Axial Vascularization

**Figure 3:**
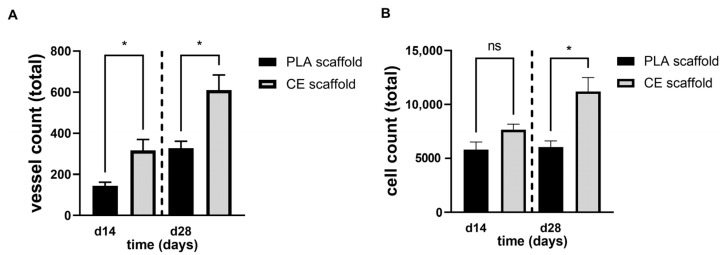
Comparison of total vessel count (**A**) and cell count (**B**) in CE- and PLA-based constructs. CE scaffolds feature a more pronounced formation of vessels and infiltrating cells on day 14 and 28 compared to corresponding PLA scaffolds, respectively. Vessel formation and cell count did increase in CE scaffolds over time, while cell count remained more stable in PLA scaffolds over time (n.s., not significant; * *p* < 0.05, comparing scaffold types).

Neovessel formation within PLA and CE scaffolds were quantified double-blind and observer-independent on day 14 and 28, respectively. While both scaffold types supported axial neovascularization and generated fully functional artificial soft-tissue flaps (Figure 2), the total number of vessels counted in CE scaffolds were higher on day 14 and day 28 compared to the respective time points in PLA scaffolds (Figure 3A, d14: 316 ± 53 and 144 ± 18, *p* < 0.05; d28: 610 ± 74 and 327 ± 34, *p* < 0.05).

While vessel ingrowth showed a continuous increase over time in both scaffolds, cell infiltration increased only in CE scaffolds (Figure 3B, *p* < 0.05). Total cell counts in CE scaffolds were generally higher compared to corresponding PLA constructs (d14: 7661.25 ± 505.93 and 5804.04 ± 716.59; d28: 11211.75 + 1278.97 and 6045.71 ± 572.72, *p* < 0.05).

**Figure 4:**
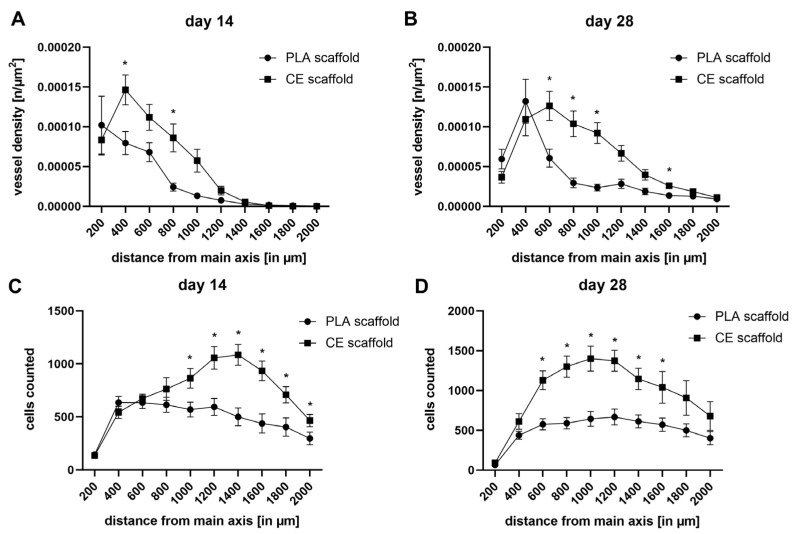
Vessel density (**A**,**B**) and cell counts (**C**,**D**) in spatial relationships to the main vessel axis. Peak vessel density shifted towards more peripheral parts of the construct over time in both scaffolds (**A**,**B**). However, both matrices showed distinct properties in terms of vessel formation (* *p* < 0.05, comparing scaffold types). Peak cell counts in CE constructs on day 28 where higher in central parts of the construct compared to PLA constructs (* *p* < 0.05, comparing scaffold types).

To focus more specific on the dynamics and spatial relationship of cell and vessel distribution, a differential analysis of subcompartments within the artificial constructs was performed. The amount of vessels and cells were analyzed in relation to their distance to the main vessel axis (vessel density in n/µm^2^ and cells counted per 200 µm diameter, see Figure 4). Maximal vessel density shifted towards more peripheral parts in the PLA constructs (distance 0–200 µm, day 14: 10.2 × 10^−5^ ± 3.6 × 10^−5^/µm^2^; distance 200–400 µm, day 28: 13.2 × 10^−5^ ± 2.8 × 10^−5^), and CE scaffolds (distance 200–400 µm, day 14: 14.6 × 10^−5^ ± 1.9 × 10^−5^/µm^2^; distance 400–600 µm, day 28: 12.6 × 10^−5^ ± 1.8 × 10^−5^) over time. While CE scaffolds exhibited a higher vessel density overall, PLA vessel density slightly increased in the peripheral areas—beyond 800 µm from the main axis—over time (Figure 4A,B). On day 14, CE scaffolds had significantly higher vessel densities between 200and 400 µm and 600 and 800 µm in relationship to the main vessel axis and between 400 and 1000 µm and 1400 and 1600 µm at the latest observation time point on day 28. Analogously, cell counts in relationship to the main vessel axis remained relatively stable for PLA scaffolds, while CE scaffolds experienced a more pronounced peak at 1000 and 1400 µm with 1401.3 ± 156.3 and 1086 ± 99.7 counted vessels on day 28 and day 14, respectively (Figure 4C,D). Peak cell counts in CE constructs were more centrally located on day 28 than on day 14. CE scaffolds had significantly higher cell counts on day 14 at distances from 800 to 2000 µm and at distances from 400 to 1600 µm on day 28.

### 2.3. Fluorescence 2D Imaging after Intravascular Perfusion of PLA-Based Constructs with MHI148-PEI

**Figure 5:**
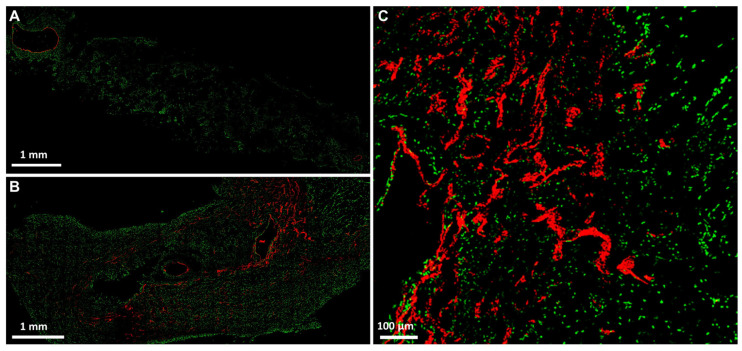
Confocal images of fresh frozen sections after MHI148-PEI perfusion (in red) with SYTOX Green staining (in green) after 7 and after 28 days, respectively (**A**–**C**). After perfusion with MHI148-PEI, vascular endothelium of main vessels, branches and capillaries (in red, **B**,**C**) were visible.

**Figure 6:**
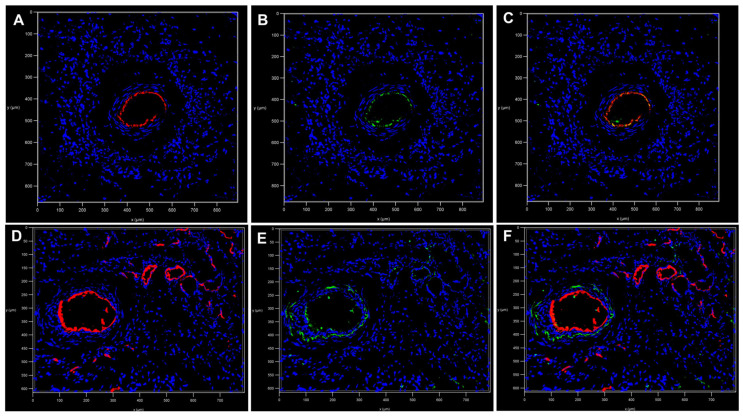
Immunostaining against CD31 (shown in green, **B**,**E**) revealed colocalization with MHI148-PEI (shown in red, **A**,**D**; merged images **C**,**F**). Main arterial inflow tract in PLA-based tissue construct after 7 days (**A**–**C**). No branches or vessel sprouts besides the arterial inflow tract were detected by CD31 staining (**B**) or intravascular MHI148-PEI (**A**) perfusion. After 28 days, vascularization of the PLA-based construct was observed by CD31 (**E**) staining as well as MHI148-PEI (**A**) perfusion.

After proving PLA scaffolds suitability for axial vascularization and soft tissue maturation by means of the AV loop technique, fluorescence dye MHI148-PEI was applied intravascularly through the constructs in conjunction with CD31 staining to validate the vascular endothelium for further downstream fluorescence imaging. After intravascular MHI148-PEI perfusion the endothelium of the main vessel axis as well as higher-order branches and smaller vessels and capillaries were adequately stained and detected, allowing for precise 2D quantitative assessments of structural features of microvascular networks within PLA-based constructs (Figure 5A–C). Immunofluorescence staining against CD31/PECAM-1 verified intravascular staining (Figure 6A–F) of MHI148-PEI with a high signal-to-noise ratio in PLA-based constructs allowing for the complete visualization of the main vessel axis, low- and high-order branches, as well as capillaries (Figure 6D–F).

## 3. Discussion

The present study demonstrated the suitability of PLA scaffolds to induce vessel sprouting originating from the AV loop, to enable soft tissue maturation, and to maintain a suitable microenvironment for axial vascularized tissue engineering. In vivo tissue engineering approaches using the AV loop technique provide an isolated and well-defined microenvironment for studies on vessel network formation and cell interaction. In the field of reconstructive microsurgery, AV loops gained popularity, and intrinsically vascularized tissues can be utilized as vascularized high-volume flaps to cover complex defects—e.g., bone-exposing defects [19,20,21,22]. In the present study, we compared both scaffold types in a histomorphometric manner following India ink perfusion. Compared to CE scaffolds, the total number of vessels in PLA scaffolds were generally lower. Furthermore, total cell counts in CE scaffolds were generally higher when compared to corresponding PLA-based constructs, while only in CE scaffolds, cell infiltration increased over time. However, cell counts in PLA-based constructs remained very stable over time. Maximal vessel density shifted towards more peripheral parts in both artificial tissue constructs over time, while PLA-based constructs peaked more centrally compared to CE-based constructs. Vessel density in PLA-based constructs remained stable in peripheral parts, while vessel density in CE scaffolds steadily declined. Cell counts in relationship to the main vessel axis remained stable for PLA scaffolds. However, CE scaffolds experienced a peak that shifted centrally over time. 

These results are in accordance with previous findings and reports on CE scaffolds and their effects on tissue vascularization. Over the last years, CE scaffolds have proven themselves as a suitable treatment option for chronic and burn wounds. CE scaffolds ideally support local tissue and blood vessel formation via intrinsic as well as extrinsic vascularization and reduce local myofibroblast formation and scar contraction [1,23,24,25,26,27,28]. PLA scaffolds, on the other hand, offer distinct advantages as a transient skin barrier and are therefore commonly used as elegant and superior treatment options in superficial and partial deep burns [29,30].

After validating the suitability of PLA scaffolds to induce axial vascularization and soft tissue formation in vivo, we perfused these constructs—via the rat aorta—with MHI148-PEI and demonstrated specificity against the vascular walls of low- and high-order branches as well as capillaries by CD31 co-staining. In contrast, CE scaffolds are more dense and lack the possibility to detect these vascular structures in the aforementioned manner. 

India ink perfusion is considered the gold standard for histomorphometrical analyses of vessel formation in artificial tissue constructs that were created in vivo. Vessels stained by means of standard histological protocols are usually easily detectable. Using automated and observer-independent quantification methods, images of histological cross sections are utilized to investigate the amount and extent of vessel sprouts in a more precise manner. Using these algorithms, we characterized the ability of CE and PLA scaffolds to induce functional microcirculatory network formation and subsequently to function as a framework for axial tissue engineering. However, intravascular perfusion with high viscosity fluids—like the India ink technique—is known to damage the endothelium and vessel walls [31,32]. Thus, these techniques impair downstream imaging and reduce the gain of information with regard to functional properties of endothelial-specific proteins and their spatial distribution within vessels. Additionally, due to the viscosity of the perfusion fluid, peripheral capillaries and smaller vessel might be constantly under-represented in histomorphometrical analyses of artificial tissue constructs. The viscosity of India ink is increased after adding gelatin and mannitol to the perfusion fluid, and the hardening of the intravascular ink leads to shrinkage and the loss of the structural integrity of vessels (as seen in Figure 2). Intact vessel walls and unscathed endothelium throughout the complete newly formed microcirculatory network is vital for comparative studies regarding morphological and functional differences of endothelial cells and their ultrastructure. Although India ink perfusion is a cost effective and reliable method for overall quantitative assessments of angiogenesis in tissue-engineered constructs, it is disadvantageous for more advanced and functional vessel visualization. 

The 3D imaging of microvascular networks was routinely performed with high-resolution µCT scans not only in artificial soft tissue constructs, but also in conventional organs [27,33]. Intravascular perfusion with a contrasting agent, like microfilm^®^, offers the fast and consistent 3D imaging of vascular networks. The 3D assessment of vascular networks via µCTs is, however, inferior to 2D histomorphometrical quantification, especially in dense and structurally heterogeneous tissue types [33].

Confocal fluorescence imaging and optical sectioning enables the 3D visualization of microcirculatory networks and facilitates advanced vessel analysis and tracking [34]. CE fibers are known to interfere with high-contrast fluorescence imaging [16]. CE scaffolds exhibit high amounts of background noise, light-scattering and autofluorescence [35,36], rendering them suboptimal for advanced fluorescence imaging and related applications.

In contrast to CE scaffolds, PLA scaffolds are homogenously designed with no CE fibers as a scaffold constituent and thus are more suited for fluorescence-based application. When these scaffolds are degraded within a bioreactor, monolactides and lactate are released, which triggers a strong angiogenetic response, leading to tissue formation and scaffold remodeling. Lactate seems to play a vital part for tissue formation as it can stabilize HIF-1alpha, the activation of VEGF and Matrix Metalloproteases [37,38,39]. Over time, as the lactate level decreases, TGF-beta activity simultaneously drops, which has been linked to reduced myofibroblast formation and scar contraction [40]. Especially in burn victims, lactate provides oxidative stress-reducing properties [41] and may inhibit a pathological cytokine release and a reduced pain sensation via inhibition of TRPV1 channels [42]. It is known that lactate, as the main constituent of these scaffolds in conjunction with oxygen, stimulates endothelial cell migration, the activity of matrix metalloproteinases, collagen synthesis and extracellular matrix formation over time [43,44,45]. The amount of connective tissue and CE content increases, both, in PLA- and CE-based constructs over time, as these tissue constructs mature in the in vivo bioreactor [27,28]. The purpose of the present study was to establish a reproducible protocol and to find a scaffold for fluorescence-based vessel imaging in axial vascularized tissue constructs and to demonstrate the suitability of fluorescence dye MHI148-PEI for the specific staining of the microcirculatory network within these constructs. Hence, the comparative analysis of CE content and formation or differences in connective tissue formation was not in the focus of this specific study. Further studies focusing on the performance of PLA and CE scaffolds in axially vascularized tissue constructs need to investigate the individual benefits and drawbacks of these scaffold types for in vivo tissue engineering.

Intravascular MHI148-PEI perfusion has previously been used for the 3D reconstruction of blood vessels maintaining the structural integrity of endothelium [46]. Using synthetic PLA scaffolds with low autofluorescence and MHI148-PEI intravascular perfusion with low viscosity, we were able to use fluorescence-based imaging techniques to visualize the microcirculatory network formation of artificially engineered soft-tissue constructs. 

Although we solely investigated 2D confocal images in this study, these findings may pave the way for more comprehensive 3D fluorescence imaging studies in artificial tissue constructs. Using specific fluorophores in combination with the clearance of tissue constructs to overcome its dense and light-scattering composition, functional and metabolic 3D imaging maps might contribute to the identification and spatial correlation of key regulators associated with axial vascularization. Optical tissue-clearing protocols in conjunction with new and innovative microscopic technologies could improve 3D fluorophore tracking and facilitate the development of automated image quantification after 3D reconstruction and the segmentation of the microvasculature within artificial constructs [47]. Expansion microscopy and immunolabeling after clearing protocols may even enable super-resolution imaging and the distribution of fluorescence dyes and antibodies [48,49]. These technologies may facilitate the characterization of key regulators within specific subdomains of axially vascularized constructs and add to the current understanding of vessel formation in tissue-engineered constructs.

## 4. Materials and Methods

### 4.1. Materials

MatriDerm (MedSkin Solutions, Dr. Suwelack AG, Billerbeck, Germany) is a commercially available bovine CE matrix in various sizes with a thickness of either 1 or 2 mm. The single-layered porous (pore size: 25–60 µm) dermal substitute is composed of native non-crosslinked collagen types I, III and V from bovine skin with 3% added alpha-elastin hydrolysate from bovine ligamentum nuchae. For the present study, we used 2 mm thick sheets. 

SupraSDRM (PolyMedics, Denkendorf, Germany) is also commercially available in different sizes with a thickness of 2 mm. As a synthetic dermal substitute, SupraSDRM is fully resorbable and composed of a polylactide copolymer, trimethylene carbonate and ε-caprolactone with pore sizes of 80–300 µm. 

### 4.2. Microsurgical Procedure and AV Loop Model

Using a surgical microscope with a 16-fold magnification (OPMI^®^ Technoskop pico, Carl Zeiss, Jena, Germany), all surgical procedures were carried out by the same investigator. 

Female Lewis rats (aged 8–12 weeks) with an average body mass of 240 g were obtained from Charles River Laboratories (Sulzfeld, Germany). The performed experiments were all in accordance with the German Animal Welfare Act and approved by the Institutional Animal Care and Use Committee of the local governmental authorities (Landesuntersuchungsamt Rheinland-Pfalz AZ G18-7-032). For analgesia, subcutaneously administered buprenorphin was used in a dosage of 0.05 mg/kg body weight (Bayer, Leverkusen, Germany). In all animals, 4 international units of heparin (Rotexmedica GmbH, Trittau, Germany) were injected via the tail vein of the animal, and 7.5 mg/kg enrofloxacin administered orally (Bayer, Leverkusen, Germany). Anesthesia was induced by 5% isoflurane (Baxter, Vienna, Austria) inhalation with a 2 L/min oxygen flow and maintained with 1.9% isoflurane and 0.6 L/min oxygen. Following hair removal, skin disinfection and surgical drape placement, a bilateral mid-ventral incision and the exposure of the femoral vascular bundle was performed. The vein was harvested over a length of 20 mm and implanted as a venous graft between the contralateral femoral artery and vein by microsurgical anastomoses with 11/0 nylon sutures (Ethilon, Ethicon, Norderstedt, Germany). After the microsurgical anastomoses were complete, 10 units of heparin were given, and vascular hemostasis was performed with a bipolar forceps (KLS Martin, Freiburg, Germany). The isolation chamber was prepared with two layers of 2 mm thick microporous polylactic acid matrix (SupraSDRM, PolyMedics, Denkendorf, Germany) or collagen–elastin matrix (MedSkin Solutions Dr. Suwelack AG, Billerbeck, Germany) embedding the AV loop as an interjacent layer, respectively. The chamber was implanted subcutaneously and sutured onto the underlying adductor fascia with 6-0 polypropylene (Prolene 6/0, Ethicon, Norderstedt, Germany). For wound closure, interrupted vertical mattress sutures with Vicryl 4-0 (Ethicon, Norderstedt, Germany) were used. Until the third postoperative day, all rats received daily buprenorphin doses (0.05 mg/kg), and on the first postoperative day, one further dose of 10 units of heparin. The rats were kept on a 12-h dark/light cycle with free access to standard chow (Sniff) and water. At the end of the experiment, the rats were sacrificed under deep anesthesia (5% isoflurane) by exsanguination, and subsequently reperfused with India Ink for comparative histomorphometrical studies.

### 4.3. Intravascular Perfusion

On day 14 and 28 after the AV loop operation, the abdominal midline was incised after the induction of anthesis and analgesia (see above). The aorta was visualized and exposed. Simultaneously, the femoral vessels of the AV Loop were exposed by a secondary inguinal incision and patency was ensured by standard microsurgical milking tests. Using a 24-gauge catheter, the aorta was cannulated, and the vascular system was washed with 100 mL of prewarmed (37 °C) isotonic salt solution containing heparin (100 IE/mL) after caval venotomy. Subsequently, the vascular system was perfused with 30 mL of prewarmed (37 °C) India ink solution (50% v/v India ink, Rohrer & Klinger, Zella-Mehlis, Germany) in 5% gelatin and 4% mannitol. MHI148-PEI perfusion was performed with 30 mL of fluorescent dye (1.5 mg/mL) diluted in aqua destillata. Near-infrared cationic dye MHI148-PEI (developed and produced at the Medical Research Center of the Medical Faculty Mannheim, Mannheim, Germany) has previously been used to visualize 3D blood vessels [50]. To enhance the binding of the fluorescent dye to the vessel wall, we clamped the femoral vessels of the AV loop for 15 min following the perfusion process. After India ink perfusion, the animal was cooled at 4° Celsius overnight before being prepared for construct explantation, which was then fixated in 4 °C cold 4% PFA. After the perfusion process with MHI148-PEI, we used perfusion fixation with 4 °C cold 4% PFA before the chamber was explanted and immediately frozen at −80 °C for 2 min.

### 4.4. Histological, Histomorphometrical and Immunofluorescence Studies

Explanted India ink-perfused AV loop constructs were formalin-fixed and paraffin-embedded. A series of three 5 µm thick tissue sections with 100 µm distance to each other were stained with hematoxylin and eosin using standard protocols. Cross-sections were obtained perpendicular to the AV loop axis and from the midpoint of the chamber between the fixation pins. Cross-sections were visualized using a motorized optical microscope with 10× magnification (Zeiss Axio Imager M2 coupled with a AxioCam HRc, Carl Zeiss, Jena, German). Histomorphometrical analysis was then conducted by using a specifically designed, user-independent and double-blind, 2-dimensional evaluation algorithm as previously described [12]. Cell counts were obtained using the open source software Ilastik [51].

For immunofluorescence studies, the MHI148-PEI perfused constructs were embedded in Tissue-Tek, and 7 µm thick tissue sections were prepared on a cryostat (CM 3050 S; Leica microsystems, Wetzlar, Germany) at −24 °C and fixed in acetone (Sigma Aldrich, St. Louis, MI, USA) at −20 °C. After a washing, permeabilization and blocking step, immunofluorescence staining against CD31 (CD31/PECAM-1 Antibody Alexa Fluor^®^ 546, Santa Cruz, CA, USA) with a dilution of 1/50 was performed at room temperature for 15 min and/or counterstained with SYTOX Green dye (dilution 1/200, Thermo Fisher Scientific, Massachusetts, United States) for 15 min. Slides were mounted in aqueous mounting medium (Abcam, Cambridge, UK). Images were obtained using a Leica SP8 confocal microscope (objective: HC PL Fluotar 16x/0.50 IMM, Leica, Wetzlar, Germany). MHI148-PEI was excited at 638 nm, and fluorescence was detected using a Cy7 emission filter. The 488 channel was used to detect autofluorescence. Image reconstructions were performed with Las X software (Leica Microsystems).

### 4.5. Statistical Analysis

Graphs were plotted with Graphpad Prism (GraphPad Software, Inc., La Jolla, CA, USA). Data are presented as means ± SEM and compared using unpaired *t*-test. For more than 2 groups, analysis of variance (one-way ANOVA) followed by the Bonferroni post hoc test was used. Differences were considered significant at a corrected error probability of *p* < 0.05.

## Data Availability

The data presented in this study are available upon request from the corresponding author.
